# Supplementary material for: Meta-Analysis and Gene Set Enrichment Relative to ER Status Reveal Elevated Activity of MYC and E2F in the “Basal” Breast Cancer Subgroup
Source: PLoS One. 2009 Mar 9;4(3):e4710. doi: 10.1371/journal.pone.0004710 (PMC2650420; doi:10.1371/journal.pone.0004710)
Supplement: Dataset S1 — GSEA results from first screen. The file contains a zipped archive of the HTML results of the second GSEA screen. Reports for enriched or depleted datasets may be accessed from the “index.html” link within the my_analysis.GseaPreranked.1219975950625.rpt.zip ZIP archive. Enrichment plots are available for each of the 50 most enriched and depleted gene sets. (13.32 MB ZIP) [file pone.0004710.s001.zip › my_analysis.GseaPreranked.1219975950625.rpt.zip!//MANALO_HYPOXIA_DN.html]

Details for gene set MANALO\_HYPOXIA\_DN[GSEA]

|  || Dataset | zSco.ERneg.to.ERpos\_collapsed |
| Phenotype | NoPhenotypeAvailable |
| Upregulated in class | na\_pos |
| GeneSet | MANALO\_HYPOXIA\_DN |
| Enrichment Score (ES) | 0.584217 |
| Normalized Enrichment Score (NES) | 2.3102846 |
| Nominal p-value | 0.0 |
| FDR q-value | 0.0 |
| FWER p-Value | 0.0 |
Table: GSEA Results Summary

  

Fig 1: Enrichment plot: MANALO\_HYPOXIA\_DN      
 Profile of the Running ES Score & Positions of GeneSet Members on the Rank Ordered List

  

| PROBE | GENE SYMBOL | GENE\_TITLE | RANK IN GENE LIST | RANK METRIC SCORE | RUNNING ES | CORE ENRICHMENT || 1 | CCNE1 |  |  | 24 | 6.716 | 0.0278 | Yes |
| 2 | MCM5 |  |  | 31 | 6.533 | 0.0562 | Yes |
| 3 | ORC6L |  |  | 33 | 6.507 | 0.0849 | Yes |
| 4 | TRIP13 |  |  | 96 | 5.890 | 0.1062 | Yes |
| 5 | CTPS |  |  | 123 | 5.706 | 0.1294 | Yes |
| 6 | SKP2 |  |  | 127 | 5.694 | 0.1544 | Yes |
| 7 | RFC3 |  |  | 164 | 5.493 | 0.1759 | Yes |
| 8 | PSME4 |  |  | 175 | 5.460 | 0.1992 | Yes |
| 9 | MCM4 |  |  | 180 | 5.436 | 0.2229 | Yes |
| 10 | MCM6 |  |  | 198 | 5.317 | 0.2451 | Yes |
| 11 | MCM10 |  |  | 201 | 5.315 | 0.2685 | Yes |
| 12 | USP1 |  |  | 222 | 5.212 | 0.2900 | Yes |
| 13 | SNRPD1 |  |  | 440 | 4.552 | 0.2937 | Yes |
| 14 | MAD2L1 |  |  | 477 | 4.446 | 0.3106 | Yes |
| 15 | NOL5A |  |  | 502 | 4.345 | 0.3280 | Yes |
| 16 | PRC1 |  |  | 543 | 4.253 | 0.3437 | Yes |
| 17 | RPL39 |  |  | 593 | 4.166 | 0.3584 | Yes |
| 18 | MCM2 |  |  | 630 | 4.080 | 0.3737 | Yes |
| 19 | DDX21 |  |  | 653 | 4.024 | 0.3898 | Yes |
| 20 | DDX11 |  |  | 654 | 4.021 | 0.4076 | Yes |
| 21 | MCM3 |  |  | 656 | 4.019 | 0.4252 | Yes |
| 22 | MRPS12 |  |  | 751 | 3.842 | 0.4351 | Yes |
| 23 | RFC2 |  |  | 762 | 3.821 | 0.4512 | Yes |
| 24 | UCHL5 |  |  | 765 | 3.814 | 0.4679 | Yes |
| 25 | CDC7 |  |  | 769 | 3.807 | 0.4845 | Yes |
| 26 | ILF3 |  |  | 801 | 3.774 | 0.4988 | Yes |
| 27 | CHAF1A |  |  | 971 | 3.525 | 0.5016 | Yes |
| 28 | USP39 |  |  | 1057 | 3.403 | 0.5102 | Yes |
| 29 | DDX18 |  |  | 1063 | 3.400 | 0.5248 | Yes |
| 30 | SNRPA1 |  |  | 1064 | 3.399 | 0.5399 | Yes |
| 31 | DNA2L |  |  | 1190 | 3.249 | 0.5448 | Yes |
| 32 | ELK1 |  |  | 1192 | 3.247 | 0.5590 | Yes |
| 33 | NOLC1 |  |  | 1222 | 3.217 | 0.5710 | Yes |
| 34 | CDC2 |  |  | 1259 | 3.182 | 0.5824 | Yes |
| 35 | GTPBP4 |  |  | 1514 | 2.931 | 0.5761 | Yes |
| 36 | TFAM |  |  | 1710 | 2.755 | 0.5736 | Yes |
| 37 | MLLT10 |  |  | 2023 | 2.510 | 0.5611 | Yes |
| 38 | RRM2 |  |  | 2047 | 2.485 | 0.5703 | Yes |
| 39 | RNU3IP2 |  |  | 2065 | 2.473 | 0.5800 | Yes |
| 40 | PPARD |  |  | 2151 | 2.417 | 0.5842 | Yes |
| 41 | SNRPD3 |  |  | 2432 | 2.233 | 0.5729 | No |
| 42 | HNRPD |  |  | 2585 | 2.156 | 0.5710 | No |
| 43 | PSMD11 |  |  | 2726 | 2.077 | 0.5696 | No |
| 44 | SNAPC5 |  |  | 2983 | 1.937 | 0.5588 | No |
| 45 | BARD1 |  |  | 3028 | 1.920 | 0.5640 | No |
| 46 | UMPS |  |  | 3124 | 1.877 | 0.5651 | No |
| 47 | CDC6 |  |  | 3185 | 1.847 | 0.5687 | No |
| 48 | CCNF |  |  | 3265 | 1.809 | 0.5707 | No |
| 49 | RPP30 |  |  | 3405 | 1.746 | 0.5679 | No |
| 50 | CCNE2 |  |  | 3424 | 1.738 | 0.5742 | No |
| 51 | PSME3 |  |  | 3489 | 1.714 | 0.5770 | No |
| 52 | RRS1 |  |  | 3719 | 1.615 | 0.5668 | No |
| 53 | PA2G4 |  |  | 3866 | 1.566 | 0.5627 | No |
| 54 | PRPS1 |  |  | 3889 | 1.556 | 0.5679 | No |
| 55 | POLG |  |  | 3925 | 1.544 | 0.5721 | No |
| 56 | POLE2 |  |  | 3975 | 1.520 | 0.5751 | No |
| 57 | HNRPU |  |  | 4024 | 1.503 | 0.5781 | No |
| 58 | RFC5 |  |  | 4034 | 1.500 | 0.5840 | No |
| 59 | RRM1 |  |  | 4872 | 1.197 | 0.5261 | No |
| 60 | POLD2 |  |  | 5531 | 0.964 | 0.4807 | No |
| 61 | ID1 |  |  | 5900 | 0.847 | 0.4567 | No |
| 62 | ZWINT |  |  | 5940 | 0.834 | 0.4574 | No |
| 63 | SFRS2 |  |  | 6677 | 0.597 | 0.4045 | No |
| 64 | TSFM |  |  | 7387 | 0.349 | 0.3525 | No |
| 65 | RB1 |  |  | 8000 | 0.105 | 0.3067 | No |
| 66 | MFN2 |  |  | 8463 | -0.061 | 0.2721 | No |
| 67 | BRCA1 |  |  | 8777 | -0.199 | 0.2493 | No |
| 68 | ZNF500 |  |  | 8832 | -0.225 | 0.2462 | No |
| 69 | HNRPDL |  |  | 9357 | -0.486 | 0.2088 | No |
| 70 | MAZ |  |  | 9415 | -0.521 | 0.2068 | No |
| 71 | MRPL46 |  |  | 9987 | -0.833 | 0.1674 | No |
| 72 | MRPL40 |  |  | 10273 | -1.004 | 0.1503 | No |
| 73 | MPHOSPH9 |  |  | 10286 | -1.019 | 0.1539 | No |
| 74 | DHX35 |  |  | 10821 | -1.405 | 0.1198 | No |
| 75 | ATIC |  |  | 12402 | -3.104 | 0.0142 | No |
| 76 | NEK4 |  |  | 12490 | -3.249 | 0.0219 | No |
| 77 | POLR3K |  |  | 12995 | -4.441 | 0.0035 | No |
| 78 | DDX23 |  |  | 13079 | -4.749 | 0.0182 | No |
Table: GSEA details [plain text format]

  

Fig 2: MANALO\_HYPOXIA\_DN: Random ES distribution      
 Gene set null distribution of ES for **MANALO\_HYPOXIA\_DN**
